# Arabidopsis *CHROMOSOME TRANSMISSION FIDELITY 7* (*AtCTF7*/*ECO1*) is required for DNA repair, mitosis and meiosis

**DOI:** 10.1111/tpj.12261

**Published:** 2013-06-10

**Authors:** Pablo Bolaños-Villegas, Xiaohui Yang, Huei-Jing Wang, Chien-Ta Juan, Min-Hsiang Chuang, Christopher A Makaroff, Guang-Yuh Jauh

**Affiliations:** 1 Institute of Plant and Microbial Biology, Academia SinicaTaipei, 11529, Taiwan; 2 Molecular and Biological Agricultural Sciences Program, Taiwan International Graduate Program, National Chung-Hsing University and Academia SinicaTaipei, 11529, Taiwan; 3 Department of Chemistry and Biochemistry, Miami UniversityOxford, OH, 45056, USA; 4 Biotechnology Center, Graduate Institute of Biotechnology, National Chung-Hsing UniversityTaichung, 402, Taiwan

**Keywords:** *CTF7/ECO1*, *Arabidopsis thaliana*, microsporocyte, microsporogenesis, chromatid cohesion, meiosis, DNA repair

## Abstract

The proper transmission of DNA in dividing cells is crucial for the survival of eukaryotic organisms. During cell division, faithful segregation of replicated chromosomes requires their tight attachment, known as sister chromatid cohesion, until anaphase. Sister chromatid cohesion is established during S-phase in a process requiring an acetyltransferase that in yeast is known as Establishment of cohesion 1 (Eco1). Inactivation of Eco1 typically disrupts chromosome segregation and homologous recombination-dependent DNA repair in dividing cells, ultimately resulting in lethality. We report here the isolation and detailed characterization of two homozygous T-DNA insertion mutants for the *Arabidopsis thaliana Eco1* homolog, *CHROMOSOME TRANSMISSION FIDELITY 7*/*ESTABLISHMENT OF COHESION 1* (*CTF7*/*ECO1*), called *ctf7-1* and *ctf7-2*. Mutants exhibited dwarfism, poor anther development and sterility. Analysis of somatic tissues by flow cytometry, scanning electron microscopy and quantitative real-time PCR identified defects in DNA repair and cell division, including an increase in the area of leaf epidermal cells, an increase in DNA content and the upregulation of genes involved in DNA repair including *BRCA1* and *PARP2*. No significant change was observed in the expression of genes that influence entry into the endocycle. Analysis of meiocytes identified changes in chromosome morphology and defective segregation; the abundance of chromosomal-bound cohesion subunits was also reduced. Transcript levels for several meiotic genes, including the recombinase genes *DMC1* and *RAD51C* and the S-phase licensing factor *CDC45* were elevated in mutant anthers. Taken together our results demonstrate that Arabidopsis *CTF7*/*ECO1* plays important roles in the preservation of genome integrity and meiosis.

## Introduction

Precise cell division with transmission of genetic information is a key process controlling growth and development in all eukaryotic organisms (Peters and Bhaskara, 2009). Chromosomes need to be properly replicated and condensed then attached to the spindle fibers in order to be distributed evenly among daughter cells (Díaz-Martínez and Clarke, 2009). The cohesin complex is critically important for these processes. Compliance with this program ensures the timely growth and development of unicellular organisms such as yeast, and the proper formation of tissues and organs in multicellular organisms such as animals and plants (Skibbens, 2010; Wu *et al*., 2010).

Proteins from the STRUCTURAL MAINTENANCE OF CHROMOSOMES (SMC) family and associated non-SMC factors are essential for the regulation of higher-order chromosomal structure in eukaryotes (Schubert, 2009). The SMC complexes are mostly composed of canonical SMC proteins, which contain a globular ATPase head, and kleisin subunits that connect the two heads to form a ring that topologically embraces nascent chromatid fibers (Peters *et al*., 2008; Watanabe, 2012). This topological entrapment allows each chromatid to be used as a template for homology-dependent DNA repair during DNA synthesis in the S-phase (Murakami *et al*., 2010), and binds sister chromatids to each other for proper spindle orientation and segregation during the G_2_/M phase (Beckouet *et al*., 2010). Chromosome cohesion involves cohesin complexes that include SMC3, SMC1, SCC3 and one of several different kleisins (Schubert, 2009). Cohesins are also important for the repair of DNA lesions caused by exposure to radiation or chemical agents post-replication, a task performed by cohesin complexes that include SMC5, SMC6A/B and the δ-kleisins NSE4A/B (Watanabe *et al*., 2009; Callegari *et al*., 2010; Kim *et al*., 2010a). Cohesins are also required for the exchange of non-sister chromatid segments between homologous chromosomes during meiosis (Kim *et al*., 2010b).

The assembly of cohesin rings around chromosomes has been extensively studied in yeast (*Saccharomyces cerevisiae*) and humans. In yeast the key regulator of cohesin establishment is an acetyltransferase known as Establishment of cohesion 1 (Eco1). Acetylation of key lysine residues K112, K113 and K84, K210 of SMC1 and SMC3, respectively, by Eco1 stabilizes the ring and facilitates binding to the α-kleisin, Sister chromatid cohesion 1 (Scc1), until anaphase (Beckouet *et al*., 2010). Then two sequential events occur, first the enzyme separase cleaves Scc1 to open the ring, followed by deacetylation of SMC1 and SMC3 by Histone lysine deacetylase 1 (Hos1) to facilitate recycling of SMC1 and SMC3 (Rivera and Losada, 2010).

In humans, point mutations in the Eco1 homolog ESCO2 lead to congenital abnormalities exemplified by Roberts syndrome (RBS). In RBS patients only 10–20% of cells show abnormal mitosis; however, all cells are hypersensitive to DNA-damaging agents and show premature centromere separation (Vega *et al*., 2005; van der Lelij *et al*., 2009; Whelan *et al*., 2012b). Recent studies on CTF7 in yeast and mouse *Eco1* and *Esco2* mutants suggest that mutations in the C-terminal acetyltransferase domain have little effect on S-phase cohesion and chromosome segregation, but increase the sensitivity to DNA-damaging agents, thereby phenocopying RBS cells (Lu *et al*., 2010; Whelan *et al*., 2012a). Mutations in the N-terminus mostly lead to defects in cohesion, and often to loss of chromosomes during mitosis (Lu *et al*., 2010; Whelan *et al*., 2012a). Moreover, in yeast it has been observed that haploid-strains defective in Eco1 are not able to sporulate, while diploid heterozygous strains are normal (Rudra and Skibbens, 2012). In mice heterozygous conditional-*Esco2* mutants show no phenotype, while homozygous embryos die at the eight-cell stage (Whelan *et al*., 2012b). These findings have led to the suggestion that Eco1 activity is dosage-dependent (Rudra and Skibbens, 2012; Whelan *et al*., 2012b), a claim made earlier by Skibbens (2010) who suggested that a decrease in yeast Eco1 activity may compromise DNA repair first and chromatid pairing second.

Nothing was known about the biological function of the *Arabidopsis thaliana* Eco1 homolog until recently, when Jiang *et al*. (2010) showed that Arabidopsis *CTF7*/*ECO1* encodes an acetyltransferase with the ability to rescue yeast *eco1* deletion mutants. Arabidopsis *CTF7*/*ECO1* encodes a 345 amino acid protein, which contains a conserved N-terminal PIP box required to interact with the replication fork subunit PROLIFERATING CELL NUCLEAR ANTIGEN (PCNA) and a zinc finger domain, important for chromatin binding. At the C-terminus of the protein is the acetyltransferase domain, required to acetylate cohesin factors (Jiang *et al*., 2010; Higashi *et al*., 2012; Rudra and Skibbens, 2012). Heterozygous *ctf7-1* mutants showed asynchronous female development, while homozygous embryos were found to arrest before or at the globular stage. Here, we report the identification and characterization of homozygous *ctf7-1* and *ctf7-2* T-DNA insertion mutants and show that CTF7/ECO1 is required to establish sister chromatid cohesion during male meiosis, and to allow proper cell division in vegetative tissues. We also show that CTF7/ECO1 is required for DNA repair and discuss these results in the context of a complex regulatory network.

## Results

### Homozygous *ctf7-1* and *ctf7-2* plants are viable but exhibit defects in vegetative and reproductive development

**Figure 1:**
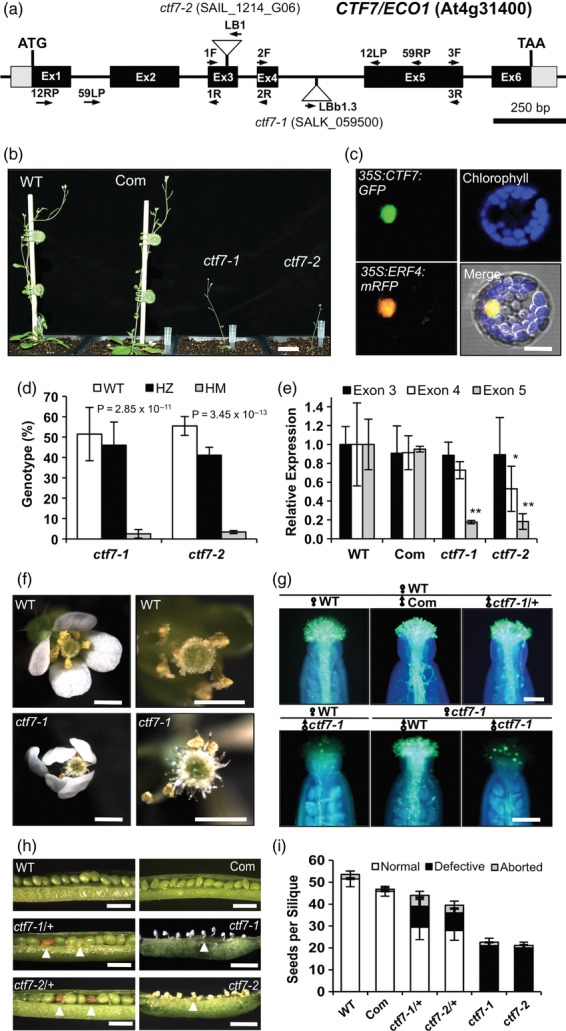
Homozygous *ctf7-1* and *ctf7-2* plants are dwarf and exhibit male sterility. (a) The diagram shows genomic organization and T-DNA insertion sites in the Arabidopsis *CTF7* locus. Dark boxes represent exons. The primer sets used for genotyping of both T-DNA lines (59LP, 59RP and LBP1.3 for *ctf7-1*; 12LP, 12RP and LB1 for *ctf7-2*) and quantitative real-time PCR (1F, 1R, 2F 2R, 3F and 3R) are indicated. (b) Homozygous *ctf7-1* and *ctf7-2* plants are dwarf and fail to develop mature siliques; however, transformation of *ctf7-1* heterozygous plants with the full genomic sequence of *CTF7*/*ECO1* allowed normal development in complementation homozygotes (Com). (c) The Arabidopsis CTF7/ECO1 protein co-localized with the ERF4 nuclear marker in leaf protoplasts. (d) Less than 4% of the progeny of self-pollinated heterozygous *ctf7-1* and *ctf7-2* (*ctf7-1*/*+, ctf7-2*/*+*) plants were homozygous (*ctf7-1, ctf7-2*). Segregation of progeny for both T-DNA alleles was non-Mendelian (not 1:3), and the respective *P*-values for the chi square test (with two degrees of freedom) were highly significant, suggesting serious developmental defects. (e) Quantitative real-time PCR experiments with primers complementary to exons 3, 4 and 5, which flank the T-DNA inserts in *ctf7-1* and *ctf7-2* indicated a significant reduction in *CTF7*/*ECO1* expression downstream of the respective T-DNA insert, while the *ctf7-1* complementation line (Com) showed values similar to wild type (WT). Results are shown as means ± SD (*n* = 3) from three biological samples. Asterisks represent significant differences (*P < 0.5, **P < 0.01; Student's *t*-test) relative to WT. (f) After anthesis, free pollen grains were easily identifiable on the surface of WT stigma, but not on *ctf7-1*. Distribution of the petals, sepals and anthers was also affected in *ctf7-1* flowers. (g) Aniline blue-stained self- and reciprocal-pollinated pistils showed normal elongation of pollen tubes from WT, *ctf7-1* heterozygous (*ctf7-1*/+), and the *ctf7-1* complementation line (Com) inside wild-type and *ctf7-1* pistils, but no seed was recovered in the latter. No elongating pollen tubes were found inside either WT or *ctf7-1* pistils after pollination with *ctf7-1* pollen grains. (h) Compared with the WT, siliques of heterozygous *ctf7-1* and *ctf7-2* plants contained a higher percentage of defective/aborted seeds. Nevertheless, *ctf7-1* and *ctf7-2* plants only produced immature siliques without normal seeds. (i) Counts of seeds per silique indicate recovery of seed development in the *ctf7-1* complementation line (Com), while *ctf7-1* and *ctf7-2* homozygotes show complete sterility. Scale bars = 1 cm for (b), 10 μm for (c), 0.5 mm for (f) and (h), 0.25 mm for (g).

It was previously shown that approximately 25% of the seed in siliques of heterozygous *ctf7-1* plants (*ctf7-1*/*+*) exhibit defects in zygote and embryo development including arrest by the early globular stage (Jiang *et al*., 2010), suggesting that inactivation of Arabidopsis *CTF7* results in embryo lethality. During the analysis of segregating populations of progeny of the *ctf7-1*/*+* (SALK_059500) and *ctf7-2*/*+* (SAIL_1214G06) T-DNA lines (Jiang *et al*., 2010; Figure 1a), we identified several slow-growing dwarf plants (Figure 1b). At about the same time analysis of the subcellular localization of AtCTF7 in Arabidopsis protoplasts indicated that AtCTF7 localizes to the nucleus (Figure 1c), a result that is in agreement with the hypothesis that Arabidopsis CTF7 is an essential nuclear protein required for growth (Jiang *et al*., 2010). Indeed, genotyping indicated that dwarf plants were homozygous for the T-DNA insert and segregated at a very low frequency (below 4%), a rate that deviated significantly from a 1:3 Mendelian ratio (Figure 1d). The phenotypes of *ctf7-1* and *ctf7-2* homozygous mutants are indistinguishable, therefore it was decided to focus efforts on the characterization of *ctf7-1*, which had been successfully complemented using the full genomic sequence of *CTF7*/*ECO1*, plus its native promoter (Jiang *et al*., 2010). This line is referred to as the *ctf7-1* complementation line (Com) in this paper. Quantitative real-time PCR (QPCR) showed that plants homozygous for either *ctf7-1* (*ctf7-1* plants) or *ctf7-2* (*ctf7-2* plants) contain <20% of wild-type (WT) *CTF7* mRNA levels corresponding to exon 5, located downstream of the corresponding T-DNA inserts (Figure 1e). Amplification of *CTF7* cDNA with primers spanning the T-DNA insert was not possible in *ctf7-1* plants, suggesting that the *ctf7-1* mutation gives rise to truncated versions of the transcript, a situation previously observed in mutations for human *ESCO2* (Vega *et al*., 2005).

Developmental defects are widespread in *ctf7-1* and *ctf7-2* seedlings, including defects in the distribution of leaves on the stem (e.g. phyllotaxy). In the WT all leaves are arranged in a spiral, while in *ctf7-1* and *ctf7-2* this arrangement shows modifications, including additional basal leaves and clusters of modified, small rosette leaves (Figure S1a). Defects in root development were also observed, including reduction in the length of the elongation zone and root swelling (Figure S1b).

The morphology of *ctf7-1* flowers was also abnormal, with enlarged papillae, defective anthers and a very limited amount of pollen (Figure 1f). Crossing experiments showed that mutant stigmas allowed the germination of WT pollen, but no mature siliques were recovered after pollination, suggesting that both male and female gametophytes are defective in *ctf7-1* (Figure 1g). No defects were observed in the germination of pollen from the *ctf7-1* complementation line or *ctf7-1*/*+* plants (Figure 1g), suggesting that *ctf7-1* heterozygous plants are not defective in the development of mature microspores.

Unlike heterozygous *ctf7-1* and *ctf7-2* plants, mature siliques of *ctf7-1* and *ctf7-2* plants were unable to develop viable seeds (Figures 1h,i). Approximately 22% of the *ctf7-1*/*+* seed reaches full size but the embryos arrest by the globular stage (Jiang *et al*., 2010); however, in *ctf7-1* and *ctf7-2* all seeds are developmentally arrested before cellularization of the endosperm (Figure 1h). Analysis of emasculated flowers suggested that unlike the case in WT siliques, unfertilized *ctf7-1* and *ctf7-2* ovules degrade within 2 days of emasculation of the anthers (Figure S1c).

### *CTF7*/*ECO1* is essential for microsporogenesis

Reciprocal pollination experiments showed that *ctf7-1* pollen is defective. Alexander staining revealed a reduction in the size and number of viable pollen grains in *ctf7-1* and *ctf7-2* anthers, which was not observed in heterozygous anthers. Pollen from *ctf7-1* and *ctf7-2* is poorly stained due to the lack of cytoplasm (Figure S2a). Staining with fluorescein diacetate (Heslop-Harrison and Heslop-Harrison, 1970) revealed that <6% of *ctf7-1* and *ctf7-2* pollen was viable, as opposed to over 85% in the WT and the complementation and heterozygous lines (*ctf7-1*/*+*, *ctf7-2*/+) (Figure S2b). Also, staining with the DNA dye 4′,6-diamidino-2-phenylindole (DAPI) indicated that <4% of *ctf7-1* and *ctf7-2* pollen fully develops into mature tricellular pollen (Figure S2c). This prompted us to examine anther and pollen development in *ctf7-1* and *ctf7-2* plants.

**Figure 2:**
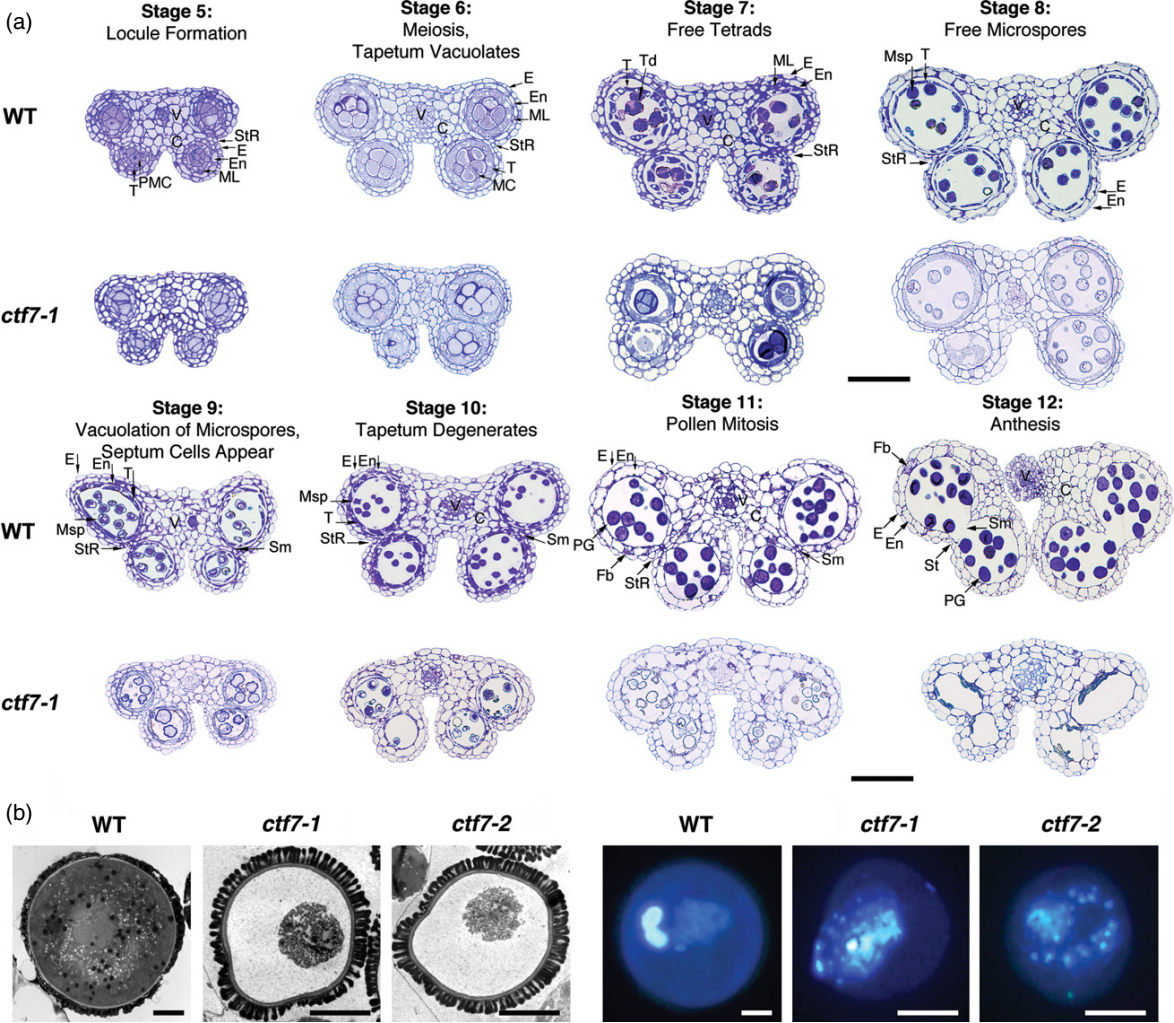
Anther dehiscence and microsporogenesis are defective in homozygous *ctf7-1* plants. (a) Sections of developing anthers revealed that *ctf7-1* had multiple anther developmental defects, including: a reduction in anther size (all stages), unsynchronized release of free tetrads (stage 7), release of irregular microspores (stage 8), lack of pollen mitosis II (stage 10) and failure of the septum (Sn) and stomium (St) to degenerate during anthesis (stage 12). (b) Left, transmission electron microscopy showed that homozygous *ctf7-1* and *ctf7-2* pollen grains were smaller, lacked cytoplasm, a vegetative nucleus and sperm cells. Right, staining with DNA dye 4′,6-diamidino-2-phenylindole (bottom) confirmed that *ctf7-1* and *ctf7-2* pollen lack identifiable vegetative nuclei and sperm cells, unlike wild-type (WT) pollen where vegetative nuclei and sperm cells were clearly observed. T, tapetum; PMC, pollen mother cell; StR, stomium regium, E, epidermis; En, endothecium; ML, middle layer; V, vascular tissue; C, connective tissue; MC, meiotic cell; Td, tetrad; Msp, microspore; Sm, septum; PG, pollen grain; Fb, fiber bands, St, stomium. Scale bars: in (a), 25 μm for stages 5–8 and 50 μm for stages 9–12; in (b) left 50 μm and right 2 μm.

Anther development in Arabidopsis starts with the formation of bilateral primordia that features locules, and vascular tissue (stages 1–4). Once the primordia are established, archesporial cells within the anther give rise to the endothecium, middle layer, tapetum and pollen mother cells (stage 5). At this stage *ctf7-1* anthers already appear smaller than in the WT (Figure 2a). Alterations in microsporogenesis were first observed during stages 6 and 7 when normally meiosis is completed and tetrads are formed. Irregular division of pollen mother cells (stage 6) and the formation of irregular tetrads (stage 7) were observed in *ctf7-1* anthers. Anthers in *ctf7-1* remained smaller than in WT as development continued, and development of the connective tissue appeared compromised as well. After the release of microspores at stage 8, the anthers of WT and *ctf7-1* continued to develop in a similar fashion; the microspores became vacuolated and the tapetum degenerated (stages 9 and 10). However, at stage 11, when microspores normally enter into mitosis and the stomium and septum degrade, *ctf7-1* microspores appeared shrunken and there was no noticeable degradation of the stomium and septum. By stage 12, WT anthers contained fully developed tricellular pollen and the anthers were undergoing anthesis. In contrast, most *ctf7-1* pollen appeared dead and no opening of the stomium and septum was observed.

Finally, severe defects in the ultrastructure of *ctf7-1* and *ctf7-2* pollen were observed, including the relative absence of an electron-dense cytoplasm, vegetative nuclei and sperm cells (Figure 2b). These results demonstrate that microsporogenesis and anthesis are defective in *ctf7* homozygous mutants.

### *ctf7-1* male meiocytes display defects in chromosome condensation, sister chromatid cohesion and the distribution of cohesin proteins

**Figure 3:**
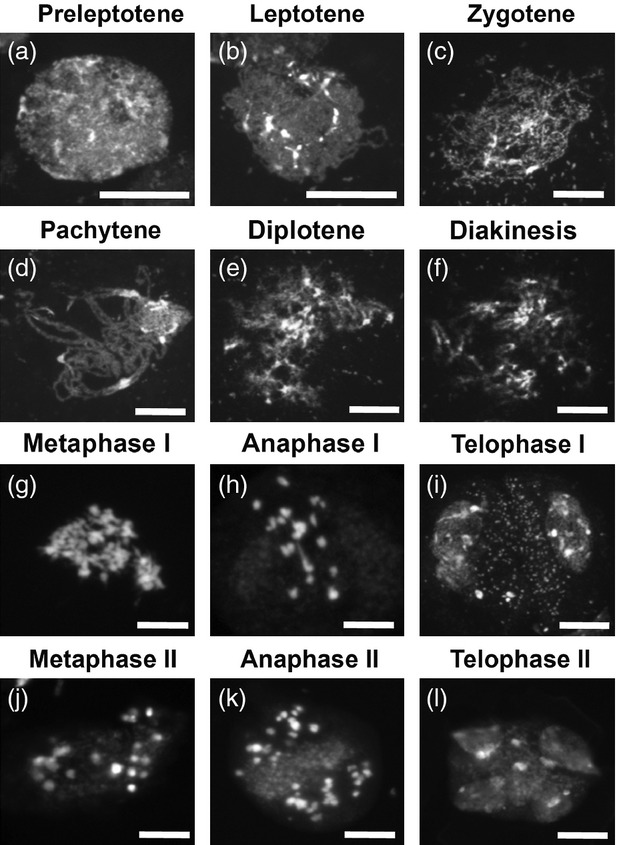
Homozygous *ctf7-1* male meiocytes are defective in chromosome pairing and segregation. 4′,6-Diamidino-2-phenylindole stained male meiocytes from *ctf7-1* plants are shown. A number of alterations are observed. (a) Pre-leptotene. Chromosomes of *ctf7-1* failed to distribute in the nuclear periphery. (b) Leptotene. Chromosomes failed to form condensed threads and remained in a scattered pattern. (c) Zygotene. Chromosomes of did not pair and align along the chromosome axis and the recombination foci were poorly observed. (d) Pachytene. Defects in synapsis with unpaired regions were observed. (e) Diplotene. Alterations in chromosome condensation with a mixture of unpaired chromosomes. (f) Diakinesis. A mixture of unpaired chromosomes, univalents and potential chromosome fragments were observed. (g) Metaphase I. Individual bivalents were not observed. Many meiocytes appeared to contain ‘extra’ chromosomes. (h) Anaphase I. Chromosomes failed to segregate properly. Lagging chromosomes and chromosome bridges were observed. (i) Telophase I. Chromosomes failed to condense properly at the poles, and lagging chromosomes were observed throughout the cell. (j) Metaphase II. Chromosomes did not align properly at the equatorial planes and remained scattered in the meiocyte. (k) Anaphase II. Chromosomes failed to segregate properly. (l) Telophase II. Polyads were observed. Scale bars = 10 μm.

Based on observations in other systems (Baudrimont *et al*., 2011; Rudra and Skibbens, 2012), we expected that inactivation of CTF7 should block the establishment of sister chromatid cohesion and result in meiotic defects. In order to investigate this possibility we analyzed meiotic chromosome spreads in *ctf7-1* plants. Alterations were observed from the earliest stages examined, with the first noticeable difference between *ctf7-1* and WT plants being the presence of fewer meiocytes overall throughout meiosis. It is not clear if this is due to the fact that the plants are smaller and less healthy, or if some *ctf7-1* microsporocytes arrest and abort prior to meiosis. Some variability was also observed in the phenotypes at different stages of meiosis, with some meiocytes appearing relatively normal; however, most meiocytes shared common phenotypes, which are described below. During pre-leptotene, WT chromosomes showed faint labeling of chromosomes with chromocenters that stain deeply (Ross *et al*., 1997; Figure S3a for WT), while in *ctf7-1* plants no chromosome axes were recognizable and the chromocenters stained very faintly (Figure 3a). Similar to WT, chromosome condensation was observed during leptotene in *ctf7-1*, although at somewhat reduced levels (Figures 3b and S3b for WT). During zygotene, chromosome alignment was reduced in *ctf7-1* (Figures 3c and S3c for WT) and ultimately a mixture of unpaired and unevenly paired chromosomes were observed at pachytene (Figures 3d S3d for WT). During diplotene, a decondensed mass of chromatin was typically observed in *ctf7-1*; no individual separated chromosomes were visible (Figures 3e and S3e for WT). In contrast to the five bivalents observed in WT at diakinesis (Mercier *et al*., 2005; Figure S3f for WT) a mixture of uncondensed chromatin, unpaired chromosomes and possibly some bivalents were observed in *ctf7-1* (Figure 3f). Beginning at diplotene and diakinesis and continuing through meiosis II, *ctf7-1* meiocytes typically appeared less condensed than their WT counterparts. A relatively small number of cells (10%) also appeared to contain extra chromosomes (Figures 3e–g and S3e–g for WT), although it is not clear whether this was caused by defects in chromosome segregation or DNA replication.

During metaphase I a mass of DNA, possibly chromosomes, congregated at the equatorial plane in *ctf7-1*; however, individual chromosomes and/or bivalents were difficult to identify (Figures 3g and S3g for WT). In contrast to WT (Figure S3h), chromosomes of *ctf7-1* did not segregate evenly at anaphase I, resulting in chromosome bridges, lagging chromosomes and a random distribution of chromosomes (Figure 3h). At telophase I in *ctf7-1* individual chromosomes could be identified while the organelle band in the equatorial region of the cells was diffuse and difficult to visualize (Figures 3i and S3i for WT). At metaphase II and anaphase II the chromosomes were irregularly scattered around the cell in *ctf7-1* (Figures 3j–k and S3j–k for WT). Finally, at telophase II, nuclear membranes formed around random groups of DNA resulting in polyads in *ctf7-1* (Figures 3i and S3i for WT).

To further investigate meiosis, and in particular sister chromatid cohesion and chromosome pairing, *in situ* hybridization was conducted with the 180 bp centromere (CEN) repeat as a probe (Armstrong *et al*., 2001) (Data S1). In leptotene, approximately 10 unpaired and well-dispersed CEN signals were observed in WT (11 ± 2, *n* = 10), while in *ctf7-1* irregular CEN signals were typically observed (Figure S4a). By zygotene, the number of CEN signals was reduced to approximately 5 ± 1 (*n* = 10) in WT. In *ctf7-1* roughly double the number of CEN foci (12 ± 1, *n* = 7) were observed, consistent with a defect in synapsis (Figure S4b). Increased CEN foci (8 ± 1, *n* = 4), were also observed during pachytene in *ctf7-1*; in addition the signals were more dispersed and less well defined than in WT, often appearing not as discrete foci but rather as long extended segments. Five easily identifiable CEN foci were observed in WT cells at diakinesis; however, 20 or more CEN signals were typically found in *ctf7-1* (Figure S4d).

During late metaphase I/early anaphase I, five pairs of CEN signals (9.1 ± 1, *n* = 10) were observed in WT, while over 20 CEN signals appeared randomly dispersed around the nucleus in *ctf7-1* (Figure S4e). By late anaphase I masses of DNA, some without CEN signals, some with two signals, and clusters of CEN signals were observed in *ctf7-1* (Figure S4f). Later in development, *ctf7-1* microspores containing varying numbers of CEN signals, some with 20 or more signals, could be observed (Figure S4g). ‘Extra’ CEN signals were also observed in interphase nuclei of some anther somatic cells of *ctf7-1* plants (Figure S4h). In contrast WT microspores and interphase anther cells always contained five and 10 CEN foci, respectively.

**Figure 4:**
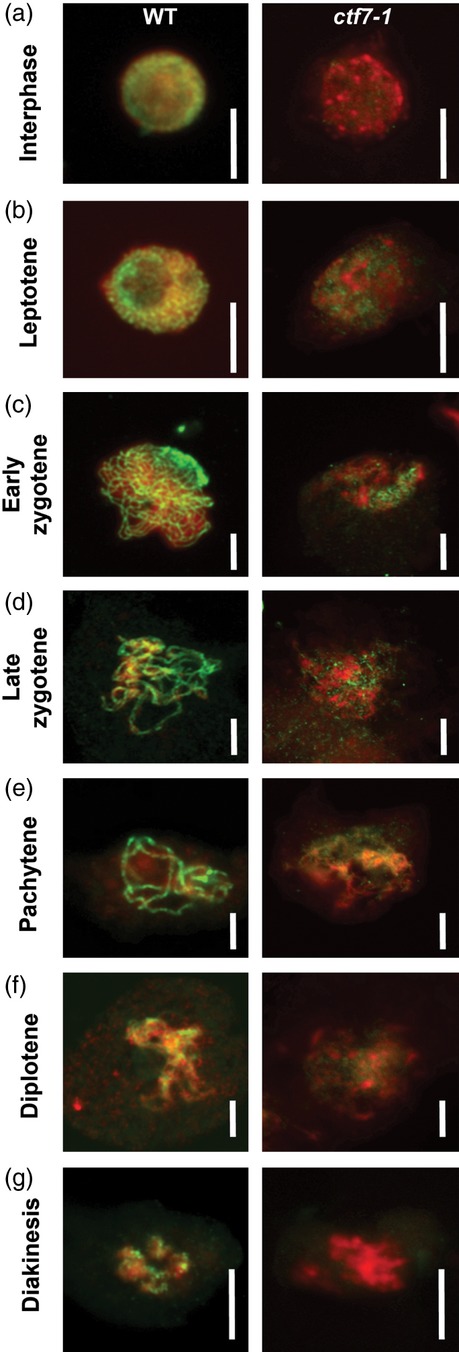
SYN1 exhibits an altered distribution pattern in homozygous *ctf7-1* male meiocytes**.** Merged images of 4′,6-diamidino-2-phenylindole stained chromosomes (red) and SYN1 (green) are shown. (a) Interphase. SYN1 is distributed throughout the nuclei of wild type (WT) male meiocytes; in contrast little or no SYN1 was observed within the nuclei of *ctf7-1*. (b) Leptotene. SYN1 decorated WT chromosome filaments as they started to condense, while in *ctf7-1* only labeling is weak and diffuse. (c), (d) Early and late zygotene. SYN1 decorates WT chromosome axes as chromosomes start to synapse. (e) Pachytene. SYN1 lined the synapsed WT bivalents, but in *ctf7-1* the labeling remained weak and diffuse. (f) Diplotene. SYN1 continues to label WT bivalents, while in *ctf7-1* both proteins appear as scattered, punctuate foci. (g) Diakinesis. Chromosome-associated SYN1 begins to weaken in WT and is absent in *ctf7-1* nuclei. Scale bars = 10 μm.

We next investigated the loading and distribution of the SYN1 and SMC3 cohesin proteins on chromosomes of *ctf7-1* meiocytes. As has been demonstrated previously, SYN1 and SMC3 display similar distribution patterns on WT meiotic chromosomes (Yang *et al*., 2011a,b; Figures 4 and S5 for WT). Diffuse nuclear labeling is observed at interphase. Beginning at early leptotene and extending into zygotene both proteins decorated the developing WT chromosomal axes. During late zygotene and pachytene the proteins lined the synapsed chromosomes. As meiosis progressed from diplotene to diakinesis the chromosome-associated cohesin signals became progressively weaker and more diffuse.

Similar to our observations in the chromosome spreading and CEN fluorescence *in situ* hybridization experiments, approximately half of the *ctf7-1* meiocytes displayed relatively normal SYN1 and SMC3 labeling patterns. However, in most cells the labeling for both proteins was very weak and irregular (Figures 4 and S5). Very little SMC3 and SYN1 signal was present in the nucleoplasm at interphase (Figures 4a and S5a for WT). Diffuse labeling of the chromatin was first observed during leptotene (Figures 4b and S5b for WT), with some labeling of thread-like structures in some cells during early zygotene (Figures 4c and S5c for WT). However, in most cases the SYN1 and SMC3 signals were diffuse and became progressively weaker as meiosis progressed into pachytene, diplotene and diakinesis stages (Figures 4d–g and S5d–g for WT).

### Several key genes for DNA repair and cell cycle progression are upregulated in *ctf7-1* plants

**Figure 5:**
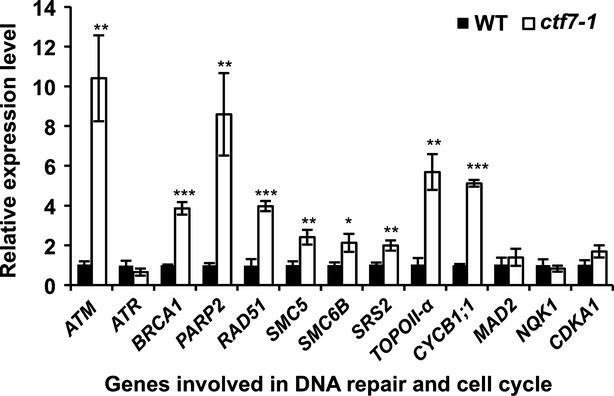
DNA-repair genes are upregulated in leaf tissue of *ctf7-1*. Complementary DNAs from 1-week-old wild-type (WT) and *ctf7-1* seedlings were generated and used in quantitative real-time PCR. Transcript levels of *ATM*, *PARP2, BRCA1, RAD51*, *SMC5*, *TOPOII-α* and *CYCB1;1* are elevated in *ctf7-1*. Data are shown as means ± SD (*n* = 3) from three biological samples. Asterisks represent significant differences (*P < 0.5, **P < 0.01, ***P < 0.001; Student's *t*-test) relative to WT.

In addition to its critical role in the establishment of sister chromatid cohesion during DNA replication, CTF7/ECO1 may be involved in DNA repair and cell cycle progression (Lu *et al*., 2010; Lyons and Morgan, 2011). We therefore investigated the effect of the *ctf7-1* mutation on the expression of a number of genes required for DNA repair and cell cycle progression. A pathway analysis using AraNet (Lee *et al*., 2010) suggested a strong functional linkage for these genes (*P* = 1.054 × 10^−82^). The relative expression levels of the selected genes were measured in triplicate through QPCR. As shown in Figure 5, large and statistically significant increases (greater than four-fold) in transcript levels were observed for several genes in *ctf7-1* plants, including: *ATM* (a kinase), *BRCA1* (a ubiquitin ligase), *PARP2* (a polymerase), *RAD51* (a gene involved in homology-dependent DNA repair), *CYCB1;1* (a G_2_/M checkpoint gene) and *TOPOII-α* (a topoisomerase) (Xie and Lam, 1994; Garcia *et al*., 2003; Preuss and Britt, 2003; Takahashi *et al*., 2010; Thomson and Guerra-Rebollo, 2010). Smaller increases (approximately two-fold) were observed for *SMC5*, *SMC6B* and *SRS2* (a helicase) (Ira *et al*., 2003; Watanabe *et al*., 2009), while no significant change was observed for *ATR* (a kinase involved in single-strand break repair) (Yoshiyama *et al*., 2009), the M-phase checkpoint genes *MAD2* and *NQK1* (Takahashi *et al*., 2010) and the gene *CDKA1,* which regulates the transition from mitosis to endocycle (Dissmeyer *et al*., 2007). Expression of other topoisomerases was not detected in either WT or *ctf7-1* samples, including *TOPOI-α*, and *TOPOI-β* (Takahashi *et al*., 2002).

**Figure 6:**
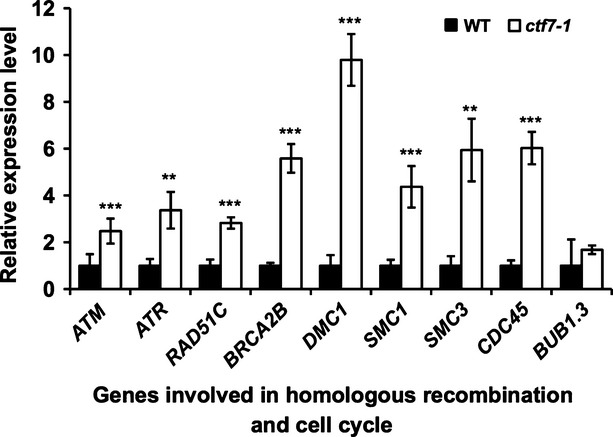
DNA recombination and canonical cohesin subunit genes are upregulated in meiocytes of *ctf7-1*. Complementary DNAs were isolated and amplified from *ctf7-1* seedlings and used in quantitative real-time PCR. Transcript levels of *ATM*, *ATR*, *RAD51C*, *BRCA2B*, *DMC1*, *SMC1*, *SMC3* and *CDC45* are elevated in *ctf7-1*. Data are shown as means ± SD (*n* = 3) from three biological samples. Asterisks represent significant differences (**P < 0.01, ***P < 0.001; Student's *t-*test) relative to wild type (WT).

Given the developmental defects observed in *ctf7-1* meiocytes the transcript level of highly expressed meiotic genes (Yang *et al*., 2011b) was measured by QPCR as well. As shown in Figure 6, statistically significant increases in transcript levels were observed for *ATM* and *ATR*, *BRCA2B*, *RAD51C*, *DMC1*, *SMC1* and *SMC3*, and *CDC45*, a gene that codes for an S-phase licensing factor and is required for meiosis (Stevens *et al*., 2004). No significant increase was observed for the mitotic checkpoint gene *BUB1.3*. Expression of several other genes was not detected, including *SRS2,* mitotic topoisomerases *TOPOII-α*, *TOPOI-α*, *TOPOI-β* (Takahashi *et al*., 2002), meiotic *TOPO III-α* (Hartung *et al*., 2008) or endoreplication factor *MYB3R4* (Takahashi *et al*., 2010).

The ability of *ctf7-1* plants to repair DNA double breaks was tested using the comet assay (Kozak *et al*., 2009) (Data S2), which has been employed in Arabidopsis mutants with defects in either chromosome cohesion or DNA repair proteins (Takahashi *et al*., 2010). Seven-day-old WT and *ctf7-1* seedlings were exposed to a bleomycin solution (50 μg ml^−1^) for 1 h and the percentage of DNA present in nuclei tails after recovery times of 0, 30 and 60 min was used to estimate the level of double-strand breaks remaining in each sample (Figure S6). In WT most double-strand breaks were repaired after 30 min and only approximately 29% remained after 1 h. In contrast approximately 79% of all double-strand breaks remained unrepaired after 1 h in *ctf7-1* plants, indicating that CTF7/ECO1 is required for DNA repair in Arabidopsis.

### *ctf7-1* and *ctf7-2* plants are defective in mitotic cell cycle progression

**Figure 7:**
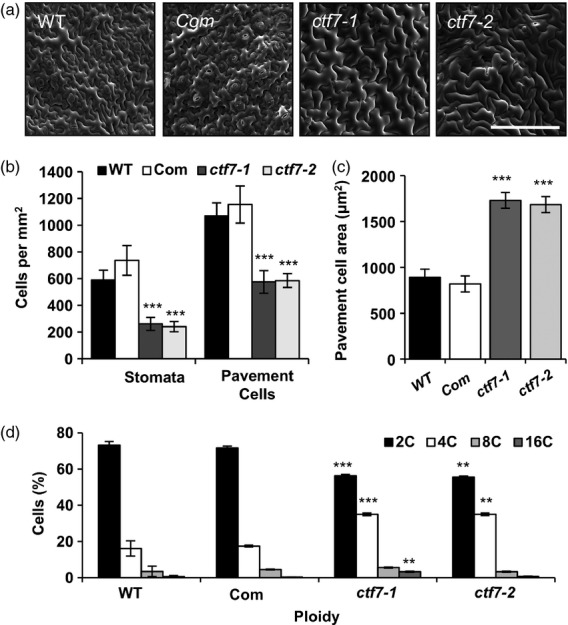
Leaves of *ctf7-1* and *ctf7-2* mutant seedlings are defective in mitotic cell division. (a) Cryo-scanning electron microscopy revealed a dramatic increase in cell size of *ctf7-1* and *ctf7-2* pavement cells (adaxial). (b) Statistical quantification of average pavement cell area. (c) Total cell number per 1 mm^2^ leaf area. (d) Flow cytometry analyses of leaf cells’ DNA content (‘C’) showed a significant increase (‘4C’) in *ctf7-1* and *ctf7-2*. Data are shown as means ± SD (*n* = 100 for B and C, and 10 000 for D) from three biological samples. Asterisks represent significant differences (**P < 0.01, ***P < 0.001; Student's *t*-test) relative to wild type (WT).

The extreme dwarf phenotype of *ctf7-1* and *ctf7-2* plants suggested that, similar to the situation in yeast (Moldovan *et al*., 2006), Arabidopsis CTF7 plays an important role in cell cycle progression. To determine if *ctf7-1* and *ctf7-2* cells show cell cycle arrest, we analyzed the morphology and density of pavement and stomata cells in the first true leaves of 7-day-old seedlings by cryo-scanning electron microscopy (cryo-SEM). In WT, pavement and stomata cells were small (Figure 7a), developed at a density of 1070 and 590 cells mm^−2^ (Figure 7b) and covered an approximate area of 892 μm^2^ (for pavement cells) and 88.0 μm^2^ (for stomata) (Figure 7c). Similar values were observed for the *ctf7-1* complementation line (Com) (Figure 7a,b). In cells of *ctf7-1* and *ctf7-2* there was a significant reduction in the density of both pavement cells (580 cells mm^−2^), and stomata (250 cells mm^−2^) (Figure 7b), accompanied by a significant increase in the average area of pavement cells (1300 μm^2^ per cell) (Figure 7c); however, no change was observed in the area of stomatal cells under cryo-SEM.

Mitotic cell cycle progression was further examined by analyzing the DNA content of leaf cells. Intact nuclei were isolated from the first leaves of 7-day-old plants, followed by flow cytometry. In WT and complementation line samples, approximately 71–73% of all nuclei had a haploid DNA content of 2, ‘2C’, which reflects normal entry into mitosis and cell division (Figure 7d). Approximately 16–17% of WT and complementation line nuclei showed a 4C value, which represents those cells that have completed DNA replication but have not entered the G_2_/M phase. In contrast, 55–56% of *ctf7-1* and *ctf7-2* nuclei showed a 4C value (Figure 7d), suggesting a defect in the ability of cells to advance into M-phase after DNA replication.

### *ctf7-1* and *ctf7-2* plants are defective in mitotic chromosome segregation

**Figure 8:**
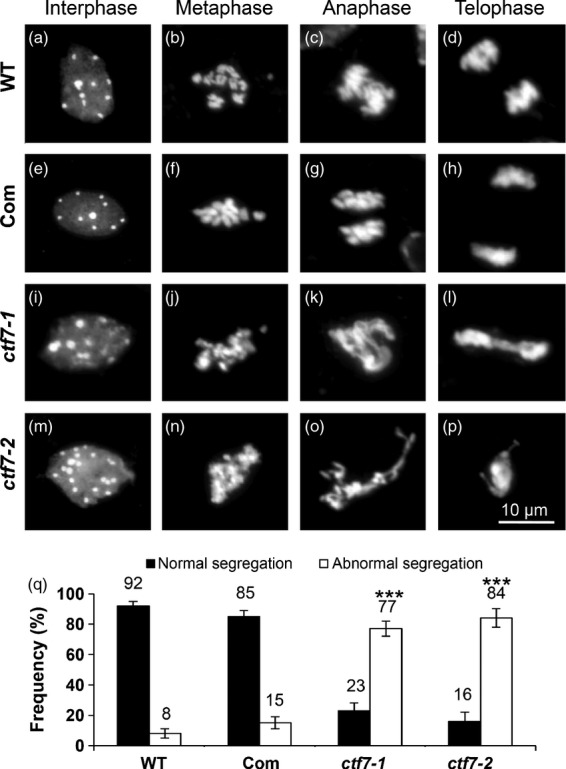
Staining with 4′,6-diamidino-2-phenylindole reveals defective chromosome segregation in homozygous *ctf7-1 and ctf7-2* mitotic root tip cells. Interphase cells in both wild type (WT) (a) and the *ctf7-1* complementation line (Com, e) show approximately 10 chromocenters corresponding to a diploid number of chromosomes. In cells of *ctf7-1* (i) and *ctf7-2* (m) the chromocenters often appeared decondensed and in some instances in excess of 10 chromocenters are observed. In metaphase, WT (b) and Com (f) cells typically show 10 condensed chromosomes; however, chromosomes in cells of *ctf7-1* (j) and *ctf7-2* (n) appear less condensed and irregularly shaped. In anaphase, WT (c) and Com (g) cells show even segregation of chromosomes, and individual chromosomes can be recognized, while chromosomes in cells of *ctf7-1* (k) and *ctf7-2* (o) appear intertwined and stretched; condensation is also defective. In telophase, WT (d) and Com (h) cell chromosomes segregate into two well-condensed masses of equal size, but in *ctf7-1* (l) and *ct7-2* (p) chromosome bridges persist and the chromosomes often recondense into a single unevenly shaped mass of DNA (l, p). Chromosome segregation in homozygous *ctf7-1 and ctf7-2* mitotic cells is statistically different from WT and shows defective chromosome segregation (q). Data are shown as means ± SD (*n* = 100) from 40 biological samples. Asterisks represent significant differences (***P < 0.001; Student's *t*-test) relative to WT. Scale bars = 10 μm.

In order to obtain direct evidence of the role of Arabidopsis *CTF7*/*ECO1* in mitotic chromosome segregation, root tips were excised from 2-week-old seedlings corresponding to WT, Com, *ctf7-1* and *ctf7-2*. As observed in Figure 8, it was possible to observe cell division from interphase to telophase. In both WT and Com, 10 chromocenters were observed during interphase (Figure 8a,e), which corresponds to a diploid number of chromosomes, while in *ctf7-1* (Figure 8i) and *ctf7-2* (Figure 8m) the chromocenters often appeared decondensed and in some cases an excess of 10 chromocenters was observed. During metaphase, WT (Figure 8b) and Com (Figure 8f) cells typically displayed 10 condensed chromosomes; however, chromosomes in cells of *ctf7-1* (Figure 8j) and *ctf7-2* (Figure 8n) appeared less condensed and irregularly shaped. At anaphase, WT (Figure 8c) and Com (Figure 8g) cells showed even segregation of chromosomes, and individual chromosomes could be recognized, while chromosomes in cells of *ctf7-1* (Figure 8k) and *ctf7-2* (Figure 8o) looked intertwined and stretched, and condensation was defective. In telophase, WT (Figure 8d) and Com (Figure 8h) cells showed segregation of chromosomes into two well-condensed masses of equal size, but in *ctf7-1* (Figure 8l) and *ctf7-2* (Figure 8p) chromosome bridges persisted and the chromosomes often recondensed into a single unevenly shaped mass of DNA (Figure 8l,p). In fact chromosome segregation in homozygous *ctf7-1* and *ctf7-2* mitotic cells is statistically different from WT (*P* < 0.001, Student's *t*-test) and shows defective chromosome segregation (Figure 8q). Taken together these results suggest that *CTF7*/*ECO1* plays a critical role in the regulation of chromosome segregation during mitotic cell division.

## Discussion

Accumulating evidence suggests that assembly of cohesion rings around nascent chromatids allows efficient DNA repair during mitosis by guaranteeing the availability of an intact template (Schubert *et al*., 2009). In yeast and mammals, a direct link exists between acetylation of cohesin rings and cohesion (Peters and Bhaskara, 2009). Acetylation of cohesin rings occurs at conserved lysine residues in SMC proteins during S phase and to a lesser extent during the G_2_/M phase (Onn *et al*., 2009). Further, establishment of cohesion requires functional interactions with subunits of the replication fork (Sherwood *et al*., 2010). In yeast this process is regulated by the acetyltransferase Eco1, which targets both SMC subunits and the PCNA subunit of the replication fork (Sherwood *et al*., 2010).

In contrast to budding yeast, very little is known about the role of Arabidopsis CTF7/ECO1. In this study we characterized the roles of Arabidopsis *CTF7*/*ECO1* in both mitosis and meiosis by characterizing plants homozygous for a T-DNA insertion in *AtCTF7*/*ECO1* (*ctf7-1* and *ctf7-2*). Given that *CTF7* is a single-copy gene in Arabidopsis and complete inactivation of *CTF7* is typically lethal, our ability to obtain plants homozygous for the T-DNA insertion was unexpected. The presence in *ctf7-1* and *ctf7-2* plants of relatively normal levels of transcript having the potential to encode the N-terminus of the protein, and reduced but detectable levels of RNA encoding the C-terminus (which comprises the acetyltransferase domain), raises the possibility that truncated forms of the protein may be produced in some cells, which allows some nuclear division. While further experiments are required to determine if partially functional forms of the protein are formed, the possibility that low levels of CTF7 activity are present in at least some cells is consistent with the fact that we observe severe defects in development and DNA repair first, and somewhat milder defects in chromosome cohesion during nuclear division. A dosage effect has been observed for cohesins in other organisms (Rudra and Skibbens, 2012), and this also appears to be the case for Arabidopsis. Plants heterozygous for *ctf7-1* and *ctf7-2* showed defects during female gametophyte development, which requires three rounds of mitosis (Figure S1c), but no developmental defects were found in microspores, which require only two rounds of mitosis (Figure S2b) (Chang *et al*., 2011). Also no obvious defects in vegetative growth were detectable in heterozygous plants, which is consistent with the hypothesis that a small reduction in acetyltransferase activity does not have a significant impact on either cohesion or cell division (Whelan *et al*., 2012a). However, *ctf7-1* homozygous plants had severe defects in male gametophytic development, including defects in anther development (Figure 2), defects in SMC3 and SYN1 binding to meiotic chromosomes (Figures 4 and S5) and dramatically reduced cohesion at centromeres (Figure S4). Alterations in cohesion distribution can affect synaptonemal complex for-mation and impair RAD51-mediated formation of chiasmata (Longhese *et al*.,2009), resulting in defective pollen formation and sterility, as we observe in *ctf7-1* (Figures 2 and 1h,i).

Homozygous *ctf7-1* and *ctf7-2* plants are dwarf (Figure 1c), suffer from cell cycle arrest (Figure 7d) and are unable to segregate chromosomes properly during mitotic cell division (Figure 8). Moreover, leaf cells from *ctf7-1* plants are unable to efficiently repair DNA double-strand breaks (Figure S6) and contain elevated levels of transcripts of genes required for double-strand break repair (Figure 5), such as the effector kinase gene *Ataxia Telangietasia Mutated* (*ATM*), polymerase *PARP2*, recombination mediator *RAD51,* cohesin subunit *SMC5* and checkpoint regulators such as *BRCA1* and *CYCB1;1* that trigger arrest at the G_2_/M phase (Garcia *et al*., 2003; Schubert *et al*., 2009; Yoshiyama *et al*., 2009). The anthers of *ctf7-1* plants contained elevated transcript levels for a number of genes, including the *ATR* kinase, the meiotic recombinase gene *DMC1* and its associated factor *RAD51C*, cohesin subunits *SMC1* and *SMC3*, *BRCA2B* and the Minichromosome Complex Maintenance (MCM) subunit gene *CDC45*, which is required for proper meiotic entry into S phase (Stevens *et al*., 2004). ATR is required for the loading of meiotic recombinases (Kurzbauer *et al*., 2012). Therefore elevated transcript levels of *ATR*, *RAD51C* and *DMC1* suggest that *ctf7-1* plants may experience DNA recombination stress. Interestingly, changes in the activity of CDC45, RAD51C and BRCA2 have all been linked to chromosomal fragmentation due to pre-replicative stress and the failure to perform homologous recombination (Stevens *et al*., 2004; Abe *et al*., 2005; Kurzbauer *et al*., 2012), a phenotype similar to what we observe in *ctf7-1* meiocytes.

Taken together, these results suggest that CTF7/ECO1 is extremely important for mitotic cell cycle progression, meiosis, mitosis and DNA repair in Arabidopsis. The dramatic developmental defects observed in *ctf7-1* plants are not observed in humans containing ESCO mutations*,* which induce defects in cohesion and DNA damage repair but not in chromosome segregation (van der Lelij *et al*., 2009). A role for ESCO2 in human meiosis has remained mostly hypothetical (Hogarth *et al*., 2011). Likewise, in *Drosophila melanogaster*, Eco1 homologs are required for checkpoint activation and chromatid cohesion (Williams *et al*., 2003), but a role in meiosis has not been demonstrated (Pimenta-Marques *et al*., 2008).

Interestingly, somatic cells of *ctf7-1* plants contained transcript levels for topoisomerase II-α, which is the only eukaryotic enzyme known to mediate topological responses to a loss of cohesion by controlling the amount of catenation in replicated DNA (Ryu *et al*., 2010). Upregulation of this gene raises the possibility that loss of CTF7-dependent cohesion may induce an increase in the activity of topoisomerase II-α as a compensatory process that restores a certain degree of cell division and allows Arabidopsis *ctf7-1* mutants to remain viable, as has been reported in human HeLa cells defective in cohesion (Díaz-Martínez *et al*., 2006). In fact, a positive reinforcing relationship between DNA catenation and cohesin-mediated cohesion has been proposed in yeast (Farcas *et al*., 2011).

Finally, some of the observed developmental defects may be the result of impaired acetylation of proteins other than SMC1 and SMC3. The Arabidopsis genome harbors several SMC-like genes that have been show to affect organ development, gene expression and chromatin compaction (Schatlowski *et al*., 2010; Moissiard *et al*., 2012) and whose relationship with CTF7/ECO1 has not yet been determined. For instance the condensin subunit SMC2A/CAP-E1 has been shown to play an important role in meiosis (Siddiqui *et al*., 2003), while condensin subunits HEB1 and -2 directly influence root and root hair development under boron stress (Sakamoto *et al*., 2011).

In conclusion we have shown that Arabidopsis *CTF7*/*ECO1* plays critical roles in meiosis, mitosis and DNA repair and is essential for microsporogenesis and anther development. While further work is required to dissect the regulatory mechanism of Arabidopsis *CTF7*/*ECO1* the availability of plants homozygous for CTF7 mutations provides a valuable system for studying this important enzyme.

## Experimental Procedures

### Plant material and growth

The WT (ecotype Columbia) and T-DNA insertional lines, SALK_059500 (*ctf7-1*), and SAIL_1214G06 (*ctf7-2*) were obtained from the Arabidopsis Biological Resource Center (Columbus, OH, USA; http://abrc.osu.edu). Seeds were surface sterilized in 30% sodium hypochlorite and germinated on half-strength Murashige and Skoog medium without sucrose, followed by stratification at 4°C for 96 h in the dark. Seedlings were grown at 21°C under a 16-h photoperiod and 60% relative humidity for approximately 5 days after the emergence of the radicle. Seedlings were then genotyped following emergence of either cotyledons or first true leaves and used for the experiments. The remaining seeds were germinated on soil to further characterize the phenotype of the mutants.

### Molecular analysis of CTF7/ECO1

The Arabidopsis *At4g31400* locus was first selected for study during a screen for potential gametophytic mutants. The T-DNA lines SALK_059500 (*ctf7-1*) and SAIL_1214G06 (*ctf7-2*) were selected in this study, since previous work showed that both independent lines display a similar phenotype and line *ctf7-1* could be successfully complemented by transformation with the Gateway binary vector pFGC5941 carrying the full genomic sequence of *At4g31400* (Jiang *et al*., 2010). Genomic DNA was extracted from segregant *ctf7-1* and *ctf7-2* T_3_ seedlings. Plants were genotyped with specific primer pairs for their corresponding T-DNA inserts and WT locus. At least five independent complementation lines were analyzed using specific primers for the *ctf7-1* insertion, for the pFGC5941 vector and for exons 3, 4 and 5 of *CTF7*/*ECO1*. Resistance to Basta, and plant fertility were also analyzed. The complete list of primers used can be found in Table S1.

### Morphological characterization of *ctf7-1* and *ctf7-2*

Images of seed set were recorded after dissection of at least 30 siliques from 7-week-old plants under a Carl Zeiss stereo Lumar V12 fluorescence stereomicroscope (Carl Zeiss, http://microscopy.zeiss.com/microscopy) connected to a Carl Zeiss AxioCam MRc5 CCD unit. Whole anther morphology was analyzed by staining with Alexander staining for 24 h at 50°C (Alexander, 1969) or by sectioning followed by staining with toluidine blue. Pollen viability was analyzed by releasing mature pollen grains into fluorescein diacetate (FDA) (Sigma-Aldrich, http://www.sigmaaldrich.com) solution for 15 min in the dark followed by observation under an Olympus BX51 epifluorescence microscope coupled to an Olympus DP70 CCD unit (Olympus, http://www.olympus-global.com/en/corc/company/lifescience). Analysis of *in vivo* pollen tube growth was performed as described by Szumlanski and Nielsen (2009) and emasculated pistils from the WT and homozygous *ctf7-1* (*ctf7-1*) were cross-pollinated and collected after 24 h. Tissue was obtained from 5-week-old plants, with three biological replicates and at least 100 pollen grains per replicate were used to estimate pollen viability.

### Analysis of subcellular localization of CTF7/ECO1

Protoplasts prepared from the leaves of 4-week old Arabidopsis plants were co-transformed with *35S:CTF7/ECO1:GFP* and the nuclear marker construct *35S:ERF4:mRFP*. Transformed protoplasts were observed by two-photon laser confocal microscopy and analyzed with the zeiss lsm image browser version 3.5.

### Analysis of ultrastructure

The ultrastructure of mature microspores was analyzed by transmission electron microscopy on a Philips CM 100 unit (Philips Research, http://www.research.philips.com/). Samples were prepared by fixation on 2.5% gluteraldehyde and 4% paraformaldehyde, in 0.1 m sodium phosphate buffer, pH 7.0 at room temperature (25°C) for 4 h. After three 20-min buffer rinses, samples were post-fixed on osmium oxide 1%. Samples were dehydrated in an acetone series, embedded in Spurr's resin, and sectioned on a Leica Ultracut E microtome (Leica Microsystems, http://www.leica-microsystems.com/). Ultra-thin sections (70–90 nm) were stained with 6% uranyl acetate and 0.4% lead citrate. Sections were observed at 80 kV. Preparation of anther sections followed the same procedure; however, semi-thin samples (1 μm) were stained instead with 10% toluidine blue in 1% Na-Borex for 1–2 min, and observed directly under a light microscope. Developmental stages were assigned according to Sanders *et al*. (1999). For the analysis of leaf cell structure, the first true leaves of 1-week-old seedlings were excised, frozen in liquid nitrogen and then transferred to a sample preparation chamber set at −160°C. After 5 min the temperature was raised to −85°C and the sample was sublimed for 15 min. After coating with platinum at −130°C the sample was transferred to a PP2000T Cryo-SEM System chamber (Quorum Technologies, http://www.quorumtechnologies.com/) and observed at −160°C on a FEI Quanta 200 scanning electron microscope (FEI, http://www.fei.com/) set at 20 kV. Results were obtained from three different biological samples containing at least three technical repeats.

### Chromosome spreads

In order to analyze male meiosis, anthers were isolated and digested as described (Yang *et al*., a,b). Following digestion, cells were transferred onto poly-l-lysine slides (Sigma-Aldrich), and covered with a cover slip. The slides were frozen on dry ice and the cover slips quickly removed. The dried slides were stained with 1.5 mg ml^−1^ DAPI (Vector Laboratories, http://www.vectorlabs.com/). In order to analyze mitosis, root tips from seedlings grown on agar were excised and placed within 200 μl PCR tubes. Fixation and digestion was performed within the tubes, and digestion was extended to 2 h.

### Antibodies and immunolocalization

SMC3 and SYN1 were localized in buds prepared as described previously (Yang *et al*., a,b). Rabbit polyclonal antibodies against SYN1 and SMC3 were then detected with Alexa Fluor 488 goat anti-rabbit secondary antibody (1:500) (Molecular Probes, http://zt.invitrogen.com/) with or without Alexa Fluor 594 goat anti-mouse secondary antibody (1:500) and observed under an epifluorescence microscope.

### Flow cytometry

Cells from fresh leaves were isolated and stained with the CyStain PI Absolute P Kit (Partec, http://www.partec.com/). Finally the nuclear suspension was run through a MoFlo XDP Laser Cell Sorter (Beckman Coulter, https://www.beckmancoulter.com/) and results were analyzed with summit V5.0 software (Beckman Coulter) from at least three different biological samples.

### Quantitative real-time PCR

Total RNA was extracted with the RNAeasy Plant Minikit (Qiagen, http://www.qiagen.com/) from WT and *ctf7-1* seedlings (1 week old), and from meiotic anthers collected from mature WT and *ctf7-1* plants. Total mRNA from meiotic anthers was further amplified with the MessageAmp II aRNA Amplification Kit (Ambion, http://zt.invitrogen.com/). First-stranded cDNA was prepared from total RNA with the Moloney murine leukemia virus reverse transcriptase system (Promega, http://www.promega.com/) according to the manufacturer's instructions. For quantitative PCR, a Power SYBR Green I Master Mix (Applied Biosystems, http://www.appliedbiosystems.com/) was used with 150–200 nm primers, 20 ng μl^−1^ cDNA and 50 μl of reverse transcriptase reaction product. Reactions were run and analyzed on the AB 7500 Real Time PCR System (Applied Biosystems). Melting curve analyses and negative controls were used to exclude primer–dimer artifacts and low specificity in the amplification. Quantitative reactions were done in triplicate and averaged. Primers specific for the 3′ end of transcripts were either designed on primer express version 3.0 (Applied Biosystems) or adapted from relevant references (Preuss and Britt, 2003; Czechowkski *et al*., 2005; Takahashi *et al*., 2010). The complete list of primers used can be found in Table S1.

## Accession Numbers

Sequence data from this article can be found in the Arabidopsis Genome Initiative or GenBank/EMBL under the following accession numbers: *At3g48190* (*ATM*), *At5g40820* (*ATR*), *At4g21070* (*BRCA1*), *At5g01630* (*BRCA2B*), *At3g19590* (*BUB3.1*), *At3g25100* (*CDC45*), *At3g48750* (*CDKA1*), *At4g31400* (*CTF7*/*ECO1*), *At4g37490* (*CYCB1;1*), *At3g22880* (*DMC1*), *At3g25980* (*MAD2*), *At5g11510* (*MYB3R4*), *At5g56580* (*NQK1*), *At4g02390* (*PARP2*), *At5g20850* (*RAD51*), *At2g45280* (*RAD51C*), *At3g54670* (*SMC1*), *At2g27170* (*SMC3*), *At5g15920* (*SMC5*), *At5g61460* (*SMC6B*), *At4g25120* (*SRS2*), *At5g55300* (*TOPOI-α*), *At5g55310* (*TOPOI-β*), *At3g23890* (*TOPOII-α*), *At5g63920* (*TOPOIII-α*).

## SUPPORTING INFORMATION

**Figure S1.** Phenotypes associated with the *ctf7-1* and *ctf7-2* homozygous mutants.

**Figure S2.** Homozygous *ctf7-1* and *ctf7-2* pollen is not viable.

**Figure S3.** Chromosome pairing and segregation patterns in wildtype meiocytes.

**Figure S4.** Chromosomes in homozygous *ctf7-1* male meiocytes exhibit defects in cohesion and chromosome fragmentation.

**Figure S5.** Cohesin subunit SMC3 exhibits an altered distribution pattern in homozygous *ctf7-1* male meiocytes.

**Figure S6.** Homozygous *ctf7-1* seedlings exhibit reduced DNA repair efficiency.

**Table S1.** Primers used in this paper.

**Data S1.** Fluorescence *in situ* hybridization in meiotic chromosomes.

**Data S2.** DNA comet assay.
