# Supplementary figures and images for: Arabidopsis CHROMOSOME TRANSMISSION FIDELITY 7 (AtCTF7/ECO1) is required for DNA repair, mitosis and meiosis
Source: Plant J. 2013 Jun 10;75(6):927–40. doi: 10.1111/tpj.12261 (PMC3824207; doi:10.1111/tpj.12261)

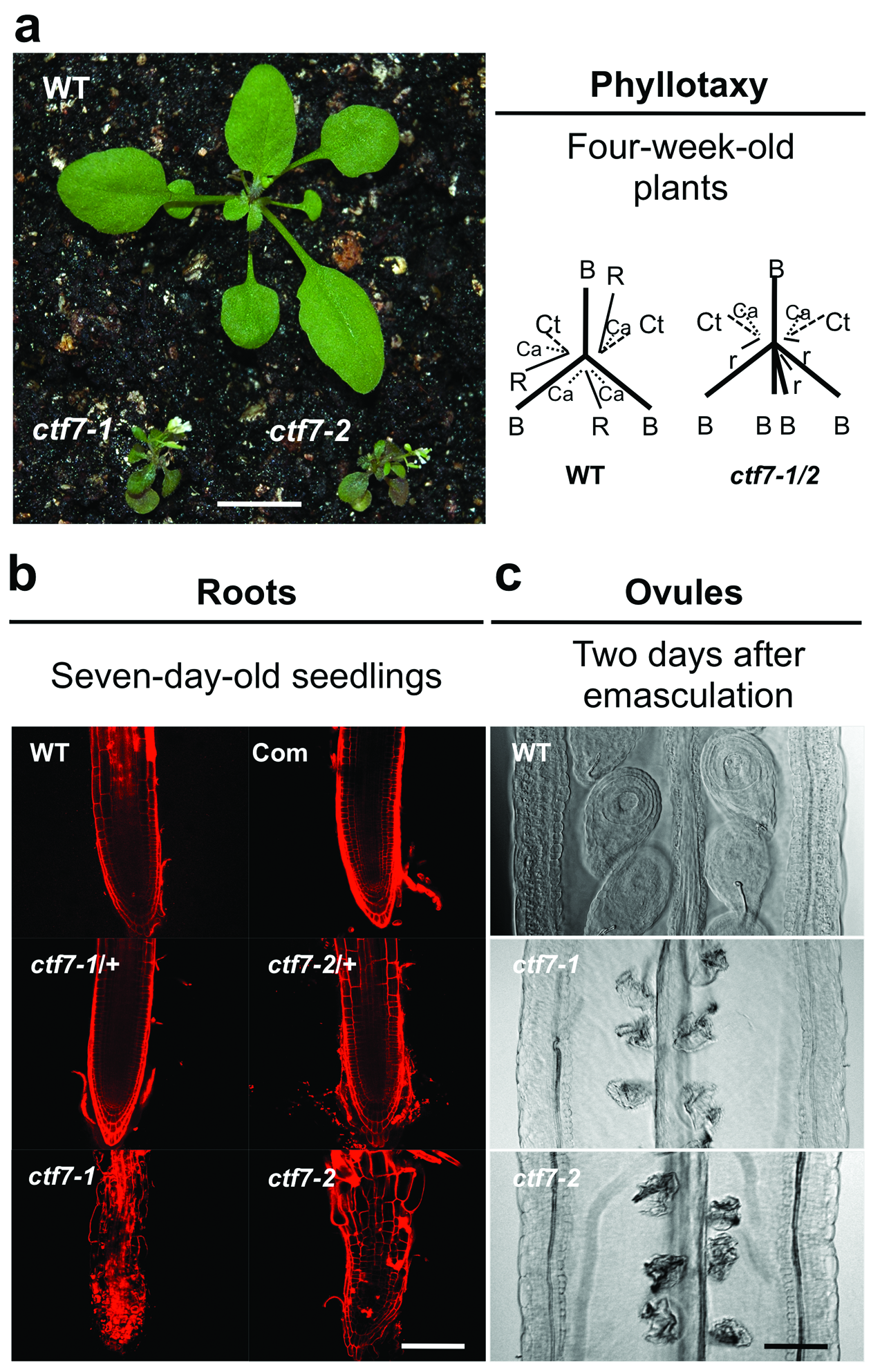

Supplement: Supplementary file 1 [file tpj0075-0927-SD1.tif]

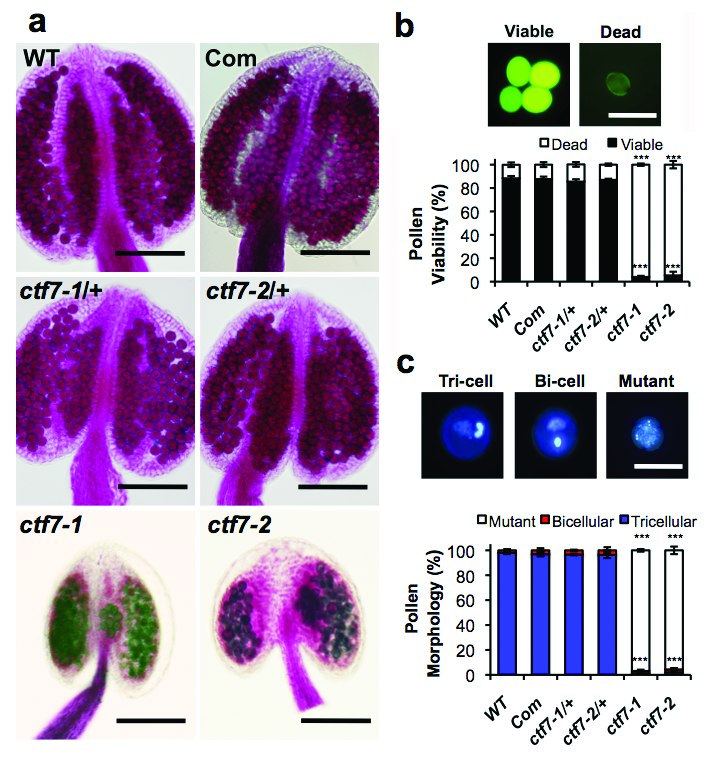

Supplement: Supplementary file 2 [file tpj0075-0927-SD2.tiff]

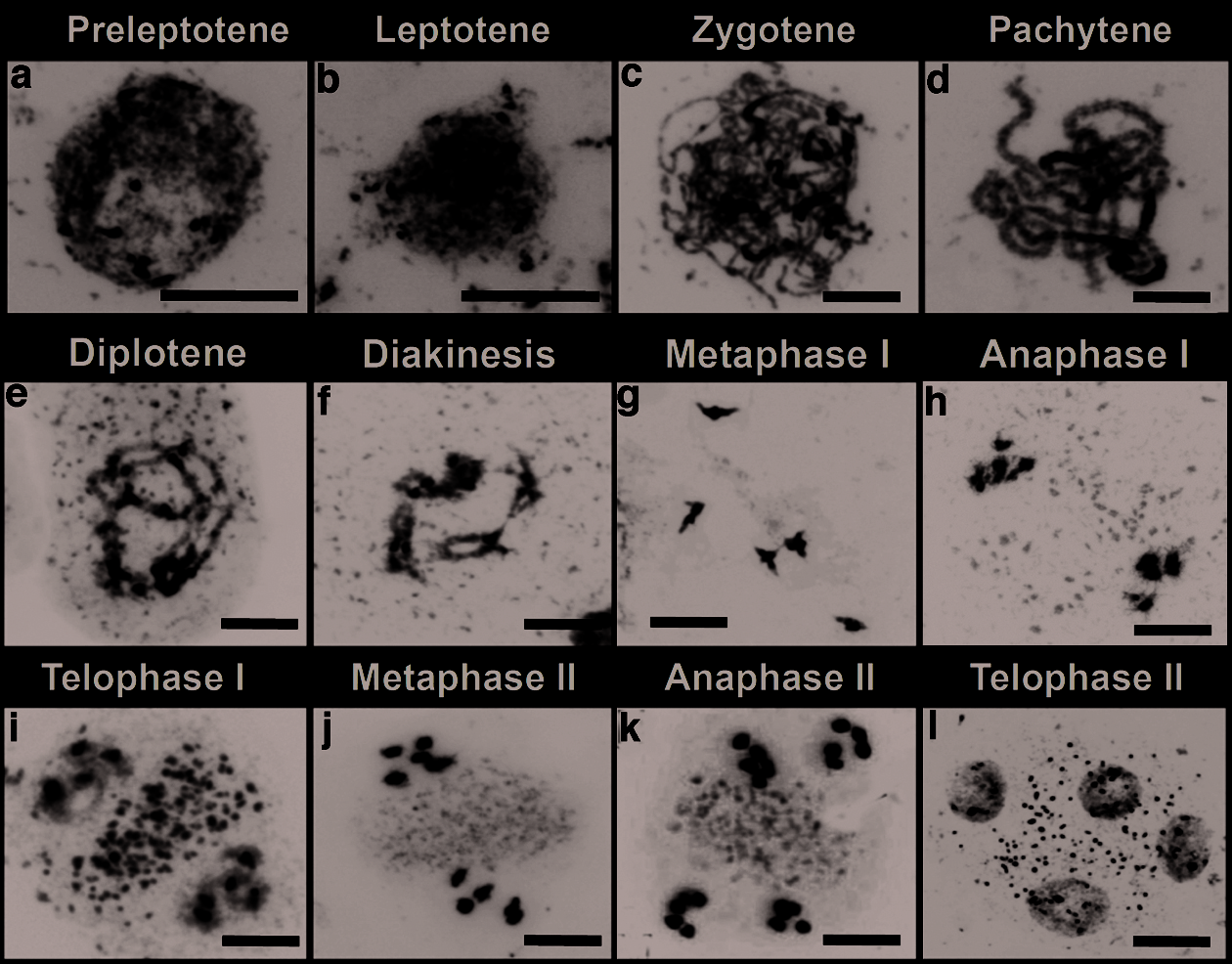

Supplement: Supplementary file 3 [file tpj0075-0927-SD3.tif]

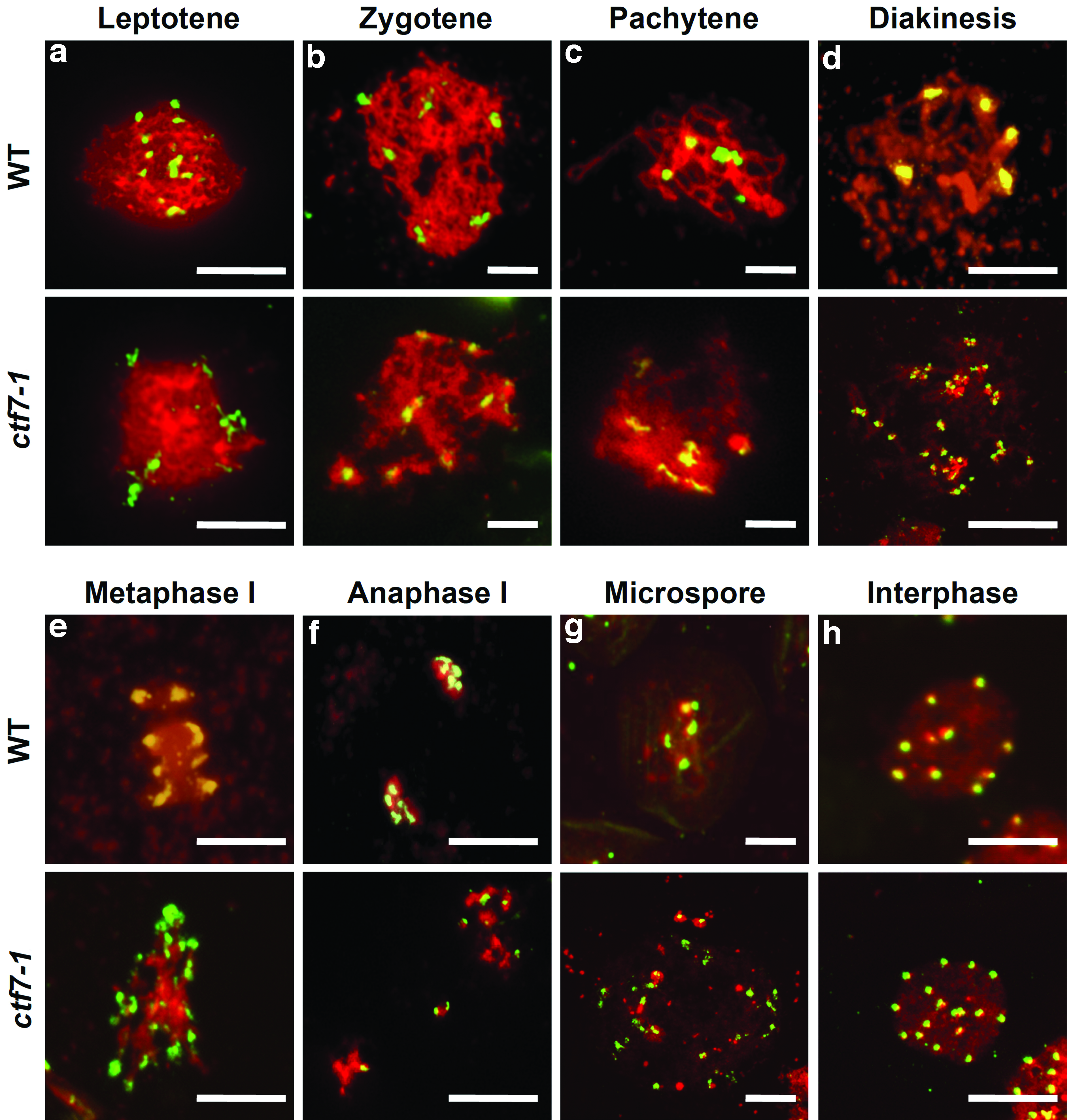

Supplement: Supplementary file 4 [file tpj0075-0927-SD4.tif]

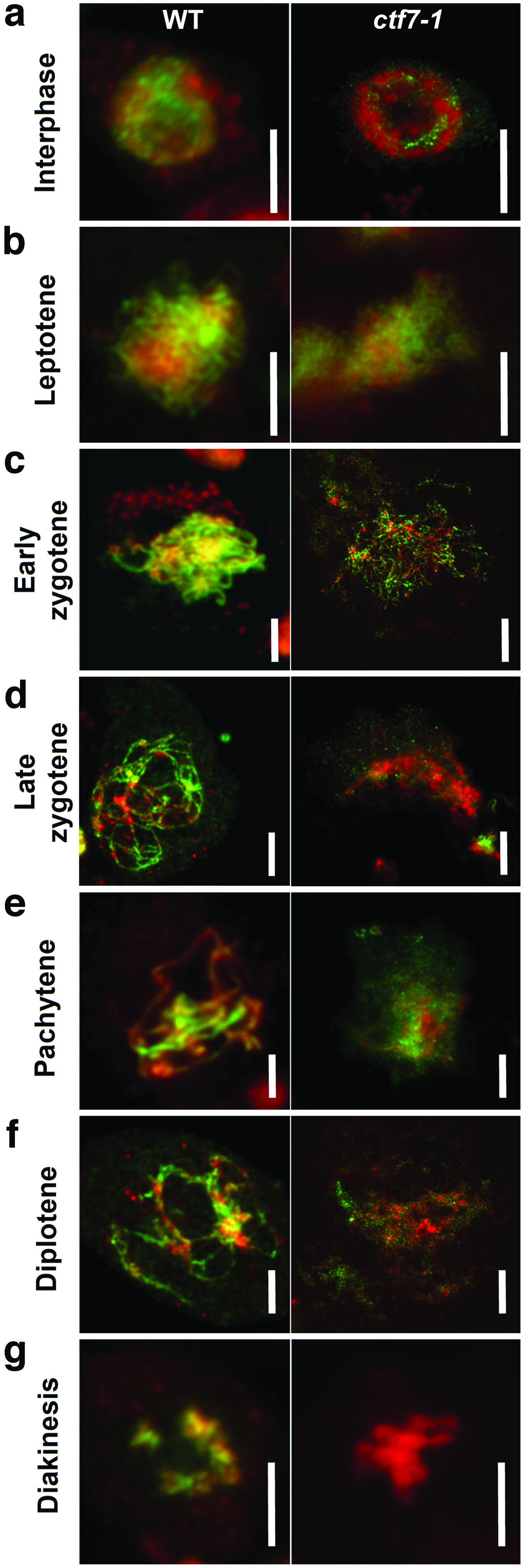

Supplement: Supplementary file 5 [file tpj0075-0927-SD5.tif]

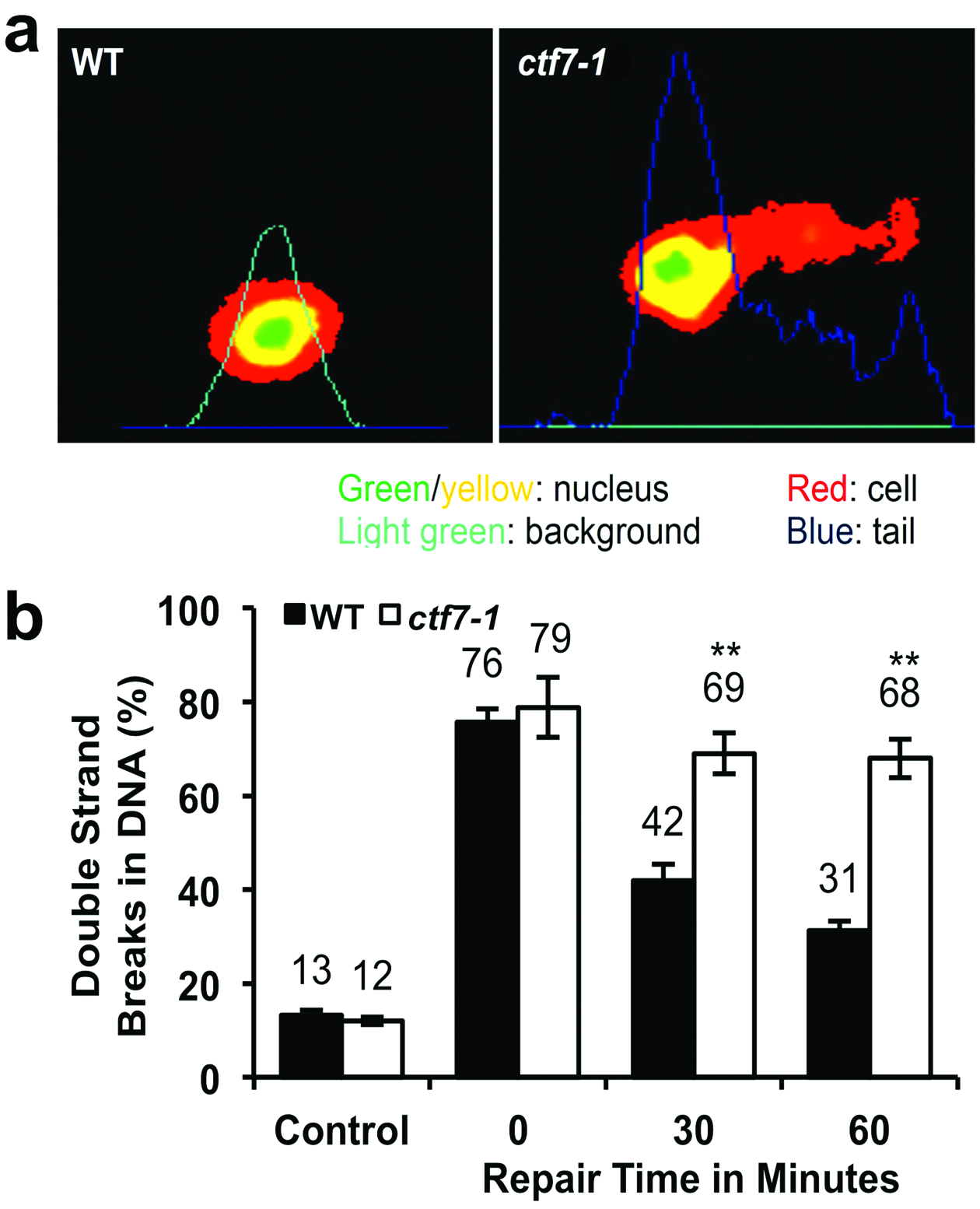

Supplement: Supplementary file 6 [file tpj0075-0927-SD6.tif]
