# Supplementary material for: Arabidopsis CHROMOSOME TRANSMISSION FIDELITY 7 (AtCTF7/ECO1) is required for DNA repair, mitosis and meiosis
Source: Plant J. 2013 Jun 10;75(6):927–40. doi: 10.1111/tpj.12261 (PMC3824207; doi:10.1111/tpj.12261)
Supplement: Supplementary file 7 [file tpj0075-0927-SD7.doc]

**Bolaños-Villegas et al.**

**Supporting Table 1. Primers used in this report.**

| Primers | Sequence 5’🡪 3’ | Purpose |
| --- | --- | --- |
| **Genotyping** | | |
| SALK059500LP | ATGAATAGAAATGAGCGATAGGC | Genomic forward |
| SALK059500RP | GAAGCTATGAGCTTAAATGCTTCC | Genomic reverse |
| SALK59RPmod | TACCCACGGCTTCAAACGTTAG | Genomic reverse for  complementation lines |
| SALK LBb1.3 | ATTTTGCCGATTTCGGAAC | Left border forward for SALK lines |
| SAIL1214G06LP | TTCCAAACTGAATGGAGGTTG | Genomic forward |
| SAIL1214G06RP | CTCCTGATTTTCTGGGTTTCC | Genomic reverse |
| SAIL LB1 | GCCTTTTCAGAAATGGATAAATAG CCTTGCTTCC | Left border forward for SAIL lines |
| BASTA-F | TTAGATCTCGGTGACGGGCA | Forward primer for  BASTA resistance gene in pFGCGW5941 complementation lines |
| BASTA-R | ACAAGCACGGTCAACTTCCGTA | Reverse primer for  BASTA resistance gene |
| **QPCR** | | |
| *CTF7 Ex3-F (1F)* | TTGCGCAGAATGTGGAGCTA | To quantify expression of individual exons within *CTF7* |
| *CTF7 Ex3-R (1R)* | GGTTCTTCTCATCTAATTCATCTCC |
| *CTF7Ex4-F (2F)* | CAAGGTGCAAGAGGTTGTGAAA |
| *CTF7Ex4-R (2R)* | CCAATCCTCACCCAACTCAA |
| *CTF7Ex5-F (3F)* | AAGAAGAAGCTAAACCAGCTGTTTGT |
| *CTF7Ex5-R (3R)* | AGGTGAGACCCAAATCGCTCTA |
| *ATM-F* | GTGCACGGCATTCGGTTTT | To quantify gene expression |
| *ATM-R* | GGAAGTTAAGCCTCGGCAATAC |
| *ATR-F* | CTATATATCCGCACCTTCGCAG |
| *ATR-R* | ACCAGACCACAGTCCTCAGTCA |
| *BRCA1-F* | TGCTAGAGCCAGAGCTGCAAG |
| *BRCA1-R* | TGGCAACCTGCAATAGAATCC |
| *BRCA2B-F* | TCACCTTAAAACCCGCAGTG |
| *BRCA2B-R* | CATGGATGACGGATTTGGAA |
| *BUB3.1-F* | AGGCCATCTTTGTAAGAAGCG |
| *BUB3.1-R* | TCGGTTTTGGTTTCACTTGG |
| *CDC45-F* | ATGTGCCTGCGCATCTCAA |
| *CDC45-R* | CATTGCCAAAAGCATTCCCT |
| *CDKA1-F* | CGCCCTGGAGCATGAATACT |
| *CDKA1-R* | TTATGCCTTTCTAAGGCATGCC |
| *CYCB1;1-F* | CCGGAACTGAATCTGCTTAGGA |
| *CYCB1;1-R* | GCTTGGTTCTTCAGCTTCTTCG |
| *DMC1-F* | AAGGCAAAGGCGATACACGTG |
| *DMC1-R* | TTCAGCGAGATTCGGAGCATC |
| *MAD2-F* | TTGATTGCTAATCCACAGATGG |
| *MAD2-R* | AAGCGTGTCGACTTTGTGAA |
| *MYB3R4-F* | GTGGCAAGCCTTCTTCACTC |
| *MYB3R4-R* | TCCTTTTCCCTGACATTTGC |
| *NQK1-F* | GCTAGCTCCATGGGACAGAG |
| *NQK1-R* | CCCCAAACTCCAAATGTCAC |
| *PARP2-F* | ATGGCGTTCTGCTCCTCTGC |
| *PARP2-F* | GGTGCTGTTTTCCCCACACC |
| *RAD51-F* | TGAGGAAAGGAAGAGCAGAGGA |
| *RAD51-R* | TCCGCTTCTGGCAAACATG |
| *RAD51C-F* | AATGGTGAAGAGCGTTACGCA |
| *RAD51C-R* | GCCGAAGCTGAAGGAAGTGAA |
| *SMC1-F* | TCCATACATAGGCCTTCACCCT |
| *SMC1-R* | GCCACGTTTAGATTATCAAGCG |
| *SMC3-F* | AGCTTGTGAGAGTCGCGGATA |
| *SMC3-R* | TCCAACGCCTGATCCTTTGAG |
| *SMC5-F* | TCCTTATATCGCGGAGCCTTC |
| *SMC5-R* | TGAGACTGCCCCAACTATCACC |
| *SMC6B-F* | CAAATGGCTGCTCCTCGTTC |
| *SMC6B-R* | CCGAGGATGCCCTTTATGG |
| *SRS2-F* | GGCAATGCATCAGAGAGTGGA |
| *SRS2-R* | TCTTGCGAGCACGAGTCATAG |
| *TOPOI-α-F* | TCGAGGTTGAGCCTCTTGTT |
| *TOPOI-α-R* | ACATCGCCATCTTTGGTTTC |
| *TOPOI-β-F* | TAGGATCACTGTTGCGTGGT |
| *TOPOI-β-R* | AACTGGCCTCAACGAACATG |
| *TOPOII-α-F* | AGGCTATAGAGGCAGCACAGAA |
| *TOPOII-α-R* | TGGTGCCTGCCTTTTAACCT |
| *TOPOIII-α-F* | ACGAATTGTCCCTCACGGG |  |
| *TOPOIII-α-R* | GCTCTCCAGTTGCAGAGAC |  |
| *ACTIN2-F* | GGCTCCTCTTAACCCAAAGGC | Expression control in vegetative tissues |
| *ACTIN2-R* | CACACCATCACCAGAATCCAGC |
| *UBQ10-F* | GGCCTTGTATAATCCCTGATGAATAAG | Expression control in meiotic pollen |
| *UBQ10-R* | AAAGAGATAACAGGAACGGAAACATAGT |
