# Supplementary material for: Arabidopsis CHROMOSOME TRANSMISSION FIDELITY 7 (AtCTF7/ECO1) is required for DNA repair, mitosis and meiosis
Source: Plant J. 2013 Jun 10;75(6):927–40. doi: 10.1111/tpj.12261 (PMC3824207; doi:10.1111/tpj.12261)
Supplement: Supplementary file 8 [file tpj0075-0927-SD8.doc]

**Supporting Experimental Procedures**

**Method S1. Fluorescence *in situ* hybridization**

To localize centromeres, fluorescence *in situ* hybridization was conducted on chromosome spreads using a probe that was prepared from the 180 bp centromeric repeat sequence (CEN), which was amplified by PCR from the pAL1 clone and labeled using the Fluorescein-High Prime DNA labeling Kit (Roche Diagnostics, www.roche-applied-science.com) according to the instructions of the manufacturer. The CEN probe was then used in hybridization solution at 5 mg/mL. Chromosomes were counterstained with DAPI and observed under an epifluorescence microscope.

**Method S2. DNA Comet Assay.**

Mutant and wild-type plants were treated with DNA-damaging agent bleomycin sulfate (Sigma-Aldrich), dissolved in liquid½ X MS medium at a concentration of 50 μg/mL, for 1 hr, then washed and places on liquid ½X MS medium without bleomycin. Cell suspension samples were prepared according to Kozak *et al*. (2009), and then processed with the Trevigen CometAssay Kit (Trevigen, www.trevigen.com) according to the instructions of the manufacturer. Slides containing the suspension were stained with SYBR Green I (Molecular Probes) and observed under an Olympus BX51 epifluorescence microscope with the FITC filter. The percent of double strand breaks per nucleus was calculated with TriTek Comet Score software version 1.5 (TriTek Corporation, tritekcorp.com), from 80 different nuclei on three different slides. The percent of damage remaining after a given repair time (t_x_) was estimated according to Kozak *et al*. (2009). Results were obtained from at least three different biological samples, with three technical repeats and a negative control.

**REFERENCES**

Kozak, J., West, C.E., White, C., da Costa-Nunes, J.A., and Angelis, K.J. (2009). Rapid repair of DNA double strand breaks in *Arabidopsis thaliana* is dependent on proteins involved in chromosome structure maintenance. *DNA Repair (Amst)***, 8,** 413-419.
