# Supplementary material for: Arabidopsis CHROMOSOME TRANSMISSION FIDELITY 7 (AtCTF7/ECO1) is required for DNA repair, mitosis and meiosis
Source: Plant J. 2013 Jun 10;75(6):927–40. doi: 10.1111/tpj.12261 (PMC3824207; doi:10.1111/tpj.12261)
Supplement: Supplementary file 9 [file tpj0075-0927-SD9.doc]

**Bolaños-Villegas *et al*.**

**Supporting Information**

**Supporting Figure 1.** Phenotypes associated to the *ctf7-1* and *ctf7-2* homozygous mutants. (a) WT plants before anthesis (approximately four-week-old) showed a normal phyllotaxy (arrangement of leaves on the stem), however in *ctf7-1* and *ctf7-2* phyllotaxy appears altered, including (but not limited) to additional basal leaves, development of small, multiple rosette leaves and loss of cauline leaves (See accompanying diagram for details). (b) Seven day-old seedlings stained with propidium iodide (PI). The WT, complementation line (Com), and heterozygous *ctf7-1* and *ctf7-2* (*ctf7-1*/+, *ctf7-2*/+) displayed roots with a clearly defined elongation zone, however roots in *ctf7-1* and *ctf7-2* showed a short elongation zone, swelling and overstaining of cells. (c) Two days after emasculation, wild-type (WT) ovules decolorized on chloral hydrate solution remained intact, but the ovules present in siliques of the *ctf7-1* and *ctf7-2* mutants collapsed. The results suggest that the ovules in *ctf7-1* and *ctf7-2* siliques were probably unfertilized. Abbreviations: B, basal leaf; Ca, cauline leaf; Ct, cotyledon; R, rosette leaf; r, modified rosette leaf. Scale bars = 1 cm in a, 100 µm in b, and 0.25 mm in c.

**Supporting Figure 2.** Homozygous *ctf7-1* and *ctf7-2* pollen is not viable.

(a) Staining of pollen with Alexander’s stain indicated presence of cytoplasm and viability in the wild-type (purple), complementation line (Com) and heterozygous *ctf7-1* and *ctf7-2* (*ctf7-1*/+, *ctf7-2*/+); however anthers of *ctf7-1* and *ctf7-2* were small and contained aborted pollen (bluish color).

(b) Staining with fluorescein diacetate (FDA) indicated that less than 6% of pollen in *ctf7-1* and *ctf7-2* were viable compared to over 85% of pollen in the wild-type, complementation line, and heterozygous *ctf7-1* and *ctf7-2*. (c) Staining with DNA dye DAPI indicated that less than of 5% of mature pollen from *ctf7-1* and *ctf7-2* has recognizable sperm cells and vegetative nuclei (tricellular and bicellular pollen). In the WT, complementation line and heterozygous *ctf7-1* and *ctf7-2* the values are over 95%. Data are shown as means ± SD (*n* = 100) from three biological samples. Asterisks represent significant differences (**P < 0.01, ***P < 0.001; Student’s *t* test) relative to WT. Scale bars = 50 µm in a, 10 µm in b and c.

**Supporting Figure 3.** Chromosome pairing and segregation patterns in WT meiocytes. DAPI stained male meiocytes from WT plants are shown.

(a) Preleptotene. Chromosomes initiate condensation, chromosome axes appear as linear and short threads, chromocenters appear as bright spots.

(b) Leptotene. Homologous chromosomes initiate alignment. (c) Zygotene. Homologous chromosomes start to synapse. (d) Pachytene. Homologous chromosomes appear well aligned and evenly paired. Synapsis is complete.

(e) Diplotene. Homologous chromosomes start to separate and discrete pairs of chromosomes (e.g. ‘bivalents’) become visible. (f) Diakinesis. Bivalents continue to condense. (g) Metaphase I. Bivalents reach maximum condensation. Five bivalents align at the equatorial plane. (h) Anaphase I. Two groups of chromosomes segregate evenly towards the opposite poles of the cells. (i) Telophase I. Chromosomes finish segregation and decondense. A clear band of organelles appears at the equatorial plane. (j) Metaphase II. Individual chromosomes align at two equatorial planes. (k) Anaphase II. Individual chromosomes initiate segregation. (l) Telophase II. Chromosome segregation is complete. Chromosomes decondense and nuclear membranes are formed around four clusters, giving rise to four haploid cells. Scale bars = 10 μm.

**Supporting Figure 4.** Chromosomes in homozygous *ctf7-1* male meiocytes exhibit defects in cohesion, chromosome fragmentation and extra chromosomes. Merged images of DAPI stained chromosomes (red) and centromere FISH signals (green) are shown for male meiocytes (a to f), microspores (g), and anther somatic cells (h).

(a) Leptotene. Approximately ten discrete CEN signals are observed in WT, while clusters of CEN signals are observed in *ctf7-1*. (b) Zygotene. CEN signals reduced to five to six in WT, while >10, were typically found in *ctf7-1*.

(c) Pachytene. Five compact CEN foci are observed in synapsed WT chromosomes. Greater than five extended foci are observed in *ctf7-1*.

(d) Diakinesis. Five CEN foci are present in desynapsing WT chromosomes. Greater than 20 small CEN signals are observed in *ctf7-1*. (e) Metaphase I. Four to five pairs of CEN signals are present in WT as bivalents attach to the spindle. Greater than 20 small CEN signals are observed in *ctf7-1*.

(f) Anaphase I. Two groups of five CEN signals are present in WT. Variable numbers of CEN signals are observed in *ctf7-1* with some “chromosomes” containing ten signals and others no CEN signals. (g) Microspore. Five CEN signals are present in WT. Variable numbers (10-20) of CEN signals are present in *ctf7-1.* (h) Interphase. WT Ten CEN signals are present in WT. Greater than ten CEN signals are present in *ctf7-1*. Scale bars = 10 μm.

**Supporting Figure 5.** Cohesin subunit SMC3 exhibits an altered distribution pattern in homozygous *ctf7-1* male meiocytes. Merged images of DAPI stained chromosomes (red) and SMC3 protein (green) are shown.

(a) Interphase. SMC3 is distributed throughout the nuclei of WT male meiocytes, in contrast little or no SMC3 was observed within the nuclei of *ctf7-1*. (b) Leptotene. SMC3 decorated WT chromosome filaments as they started to condense, while in *ctf7-1* only labeling is weak and diffuse.

(c and d) Early and Late Zygotene. SMC3 decorates WT chromosome axes as chromosomes start to synapse. SMC3 also showed some labeling of the developing axes during early zygotene. (e) Pachytene. SMC3 lined the synapsed WT bivalents, but in *ctf7-1* the labeling remained diffuse. (f) Diplotene. SMC3 continues to label WT bivalents, while in *ctf7-1* the protein appear as scattered, punctuate foci. (g) Diakinesis. Chromosome-associated SMC3 begins to weaken in WT and is absent in *ctf7-1* nuclei. Scale bars = 10 μm.

**Supporting Figure 6.** Homozygous *ctf7-1* seedlings exhibit reduced DNA repair efficiency.

(top) Electrophoresis of leaf nuclei isolated from one-week-old plants after exposure to the radiomimetic agent, bleomycin sulfate. The images were processed with software to estimate the length of DNA tails, a sign of DNA damage. Compared to the wild-type (WT), the efficiency of DNA repair was dramatically reduced in *ctf7-1*. (bottom) The length of DNA tails was used to calculate the percent of double strand breaks remaining on nuclei after 0, 30 and 60 minutes exposure to bleomycin. After one hour, wild-type nuclei efficiently repaired most of the damage and left about one third (29.6 ± 1.51 %) of the initial double strand-breaks unrepaired. In contrast *ctf7-1* nuclei still exhibited substantial damage leaving approximately three quarters (76.5 ± 9.73 %) of all breaks unrepaired. Data are shown as means ± SD (*n* = 80) from three biological samples. Asterisks represent significant differences (**P < 0.01; Student’s *t* test) relative to WT.
